# Exploring the interplay of parenting styles, basic empathy, domestic violence, and bystander behavior in adolescent school bullying: a moderated mediation analysis

**DOI:** 10.3389/fpsyt.2024.1452396

**Published:** 2024-09-09

**Authors:** Lujie Zhong, Yutong Ying, Chunni Zeng, Jiaying Li, Yun Li

**Affiliations:** School of Health Management, Guangzhou Medical University, Guangzhou, China

**Keywords:** parenting styles, basic empathy, family violence, bystander behavior, school bullying, adolescents, mediation

## Abstract

**Introduction:**

This study investigates how parental styles, basic empathy, and family violence influence adolescents’ bystander behaviors in school bullying.

**Methods:**

A survey was conducted with 1,067 students from three middle schools in southern China. Multifactor logistic regression and a moderated mediation model were employed to analyze the relationships between positive and negative parental styles, basic empathy, and bystander behaviors.

**Results:**

The study found significant correlations and predictive relationships: Positive parental styles were strongly associated with increased basic empathy (*r* = 0.29, *p* < 0.01) and behaviors that protect victims (*r* = 0.29, *p* < 0.01). In contrast, negative parental styles correlated positively with behaviors that support bullying (*r* = 0.12, *p* < 0.01) and instances of family violence (*r* = 0.62, *p* < 0.01). Basic empathy negatively predicted behaviors that promote bullying (*β* = -0.098, *p* < 0.01) and positively predicted protective behaviors toward victims (*β* = 0.249, *p* < 0.001). Furthermore, family violence weakened the positive effects of positive parental styles on both empathy (*β* = -0.075, *p* < 0.001) and protective behaviors (*β* = -0.025, *p* < 0.01).

**Conclusion:**

The findings indicate that positive parental styles indirectly promote adolescents’ victim protector behaviors by enhancing their basic empathy, underscoring the importance of emotional cultivation. Meanwhile, family violence weakens the positive impact of these parental styles on basic empathy and protective behaviors, harming adolescents’ emotional security and behavioral norms.

## 1 Introduction

Bullying is defined as the repeated and prolonged exposure of a student to negative actions from one or more other students (1). This behavior is characterized by its persistence, repetition, and hidden nature, and it often continues over an extended period. A global survey on adolescent health behaviors conducted by the United Nations Children’s Fund (UNICEF) and the World Health Organization (WHO) revealed that approximately one-third of students worldwide experienced bullying at least once in the past 30 days (2). Bullying has significant impacts on both perpetrators and victims. Perpetrators may experience cognitive and behavioral disturbances, leading to psychological distortions and antisocial personality issues (3, 4). Victims, on the other hand, may struggle to concentrate in class, skip school, experience low self-esteem, psychological trauma, and even engage in self-harm or suicide (5, 6). According to previous research (7–9), bystanders are also crucial players in the phenomenon of school bullying. This study distinguishes bystander behavior in school bullying into three categories: bully promoter, who directly or indirectly supports or encourages bullying behavior; victim protector, who takes proactive actions to intervene or shield the victim; and outsider, who chooses not to take action or remains passive. Many intervention programs have proven effective in reducing bullying incidents by providing systematic training to bystanders (10–12). Still, further research on the influencing factors and intervention strategies of bystander behavior is of significant importance in reducing the occurrence of school bullying.

Parental parenting style is a significant factor influencing school bullying, as it is closely related to adolescent behavioral problems (13, 14). When parents provide sufficient support and warmth during their upbringing, it typically promotes positive interactions and emotional support among family members, making adolescents more likely to engage in prosocial behavior, such as helping others in crisis situations (15). An emotionally warm parenting style benefits early emotional and social development (16), fostering higher self-esteem, psychological well-being, and social skills in children (17), while also reducing the occurrence of psychological health problems (18), thereby increasing the likelihood of positive actions to help victims of school bullying. However, low levels of parental support are significantly negatively correlated with internalizing problems in adolescents, such as anxiety and depression (19). Additionally, rejecting and overprotective parenting styles often create tense family atmospheres and negative emotions, which can lead to more psychological health problems (20). In such environments, adolescents may experience fear, anxiety, or depression. Rejecting parenting styles can contribute to the development of negative emotional responses, potentially increasing their likelihood of engaging in bullying behaviors (20). Overprotective parenting styles may result in adolescents lacking problem-solving abilities, making them passive and indifferent to bullying behavior (21). Moisuc et al. found that proactive bystander behavior in protecting victims is associated with individual traits such as social responsibility, perseverance, and altruism, which can be effectively fostered by an emotionally warm parental parenting style (22). Similarly, Iotti et al. discovered that authoritative parenting is positively correlated with adolescents’ prosocial behaviors, particularly their motivation to advocate for bullying victims (23). These findings suggest that an emotionally supportive parenting style can significantly enhance positive social behaviors in adolescents. Conversely, previous studies have indicated that the parenting style received by victims of bullying is often overly protective (11), rejecting parenting styles are significantly negatively correlated with adolescent prosocial behavior and emotional regulation abilities (24), and children raised with a rejecting parenting style are more likely to become bullying targets (25). Thus, while research on the relationship between parental parenting styles and bullying behavior has been extensive, studies on bystander behavior remain relatively scarce. Therefore, this study focuses on exploring the influence of different parental parenting styles on bystander behavior among adolescents in school contexts.

Thus, while research on the relationship between parental parenting styles and bullying behavior has been extensive, studies on bystander behavior remain relatively scarce. Therefore, this study focuses on exploring the influence of different parental parenting styles on bystander behavior among adolescents in school contexts.

Empathy is “an individual’s emotional response based on an understanding of another person’s emotional state or condition, which is equivalent to or similar to what others are experiencing or may experience” (26). Empathy is generally defined from two dimensions: cognitive empathy refers to the ability to communicate, tolerate, identify, and perceive emotions, while affective empathy refers to the ability to perceive and share others’ positive and negative emotions (27, 28). Low empathy is a contributing factor to the development of violent behavior (29). Research indicates that empathy is negatively correlated with violent or aggressive behavior, i.e., the higher the empathy level, the lower the probability of violent or aggressive behavior, while lower empathy levels are associated with higher probabilities of violent or aggressive behavior (30). Thus, empathy is also an important influencing factor in bystander behavior.

It is worth noting that adolescents who have experienced family violence are more likely to become victims of school bullying (31–33). The occurrence of domestic violence increases the likelihood of adolescents engaging in a range of health risk behaviors (34), with school bullying representing a significant form of these health risk behaviors. A study in Italy found that among adolescents who experienced parental family violence, 71% experienced school bullying, while among adolescents who did not experience parental family violence, 56.9% experienced bullying, with a statistically significant difference between the two groups (35). A study in China found that experiencing family violence is a predictive factor for being bullied (36). Additionally, related research also indicates that adolescents who witness violence between their parents are positively correlated with their own experiences of being bullied at school (37). Furthermore, family violence can adversely affect family communication, which has a significant negative correlation with adolescents’ anxiety and depression (38). This psychological distress can, in turn, impair adolescents’ social interactions within the school environment. Moreover, the presence of family violence increases the vulnerability of adolescents to becoming victims of school bullying.

Previous research has primarily focused on the relationship between parental parenting styles and school bullying, along with the negative effects of bullying on adolescents’ psychological health. However, studies on the roles of empathy and family violence in school bullying, particularly concerning bystanders, remain limited. This study addresses these gaps by using mediation-moderation analysis to explore the connections between parental parenting styles, empathy, and bystander behavior among adolescents. The study hypothesizes that positive parenting styles are positively related to empathy skills (H1), which, in turn, are positively correlated with victim protection behavior (H2). Furthermore, empathy skills are posited to mediate the relationship between parenting styles and victim protection behavior (H3), while family violence may moderate these relationships (H4). The findings aim to offer scientific insights for the prevention and intervention of adolescent school bullying.

## 2 Materials and methods

### 2.1 Participants

**Figure 1:**
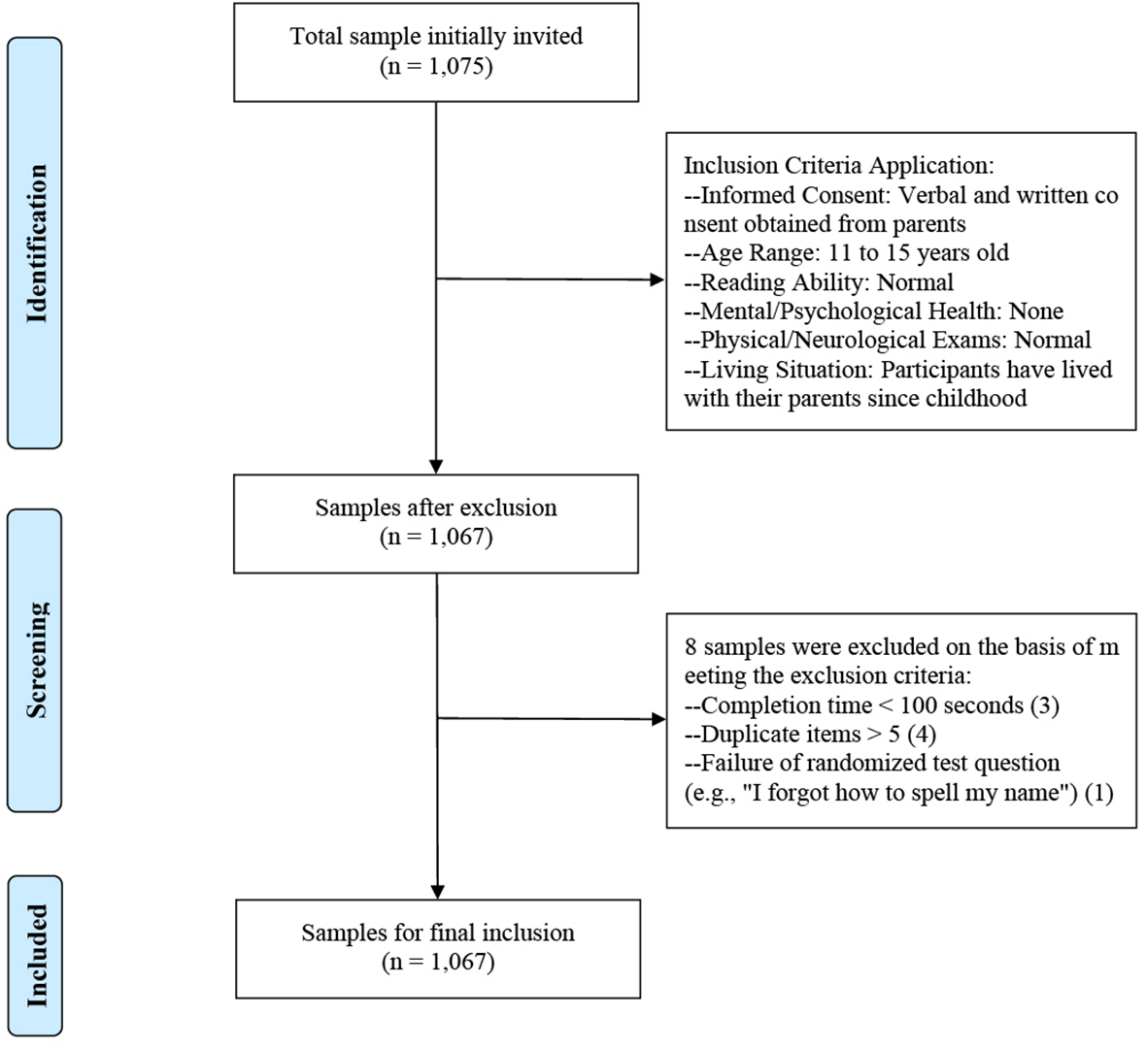
Flow chart for the inclusion and exclusion of participants.

This study conducted a survey in three middle schools in southern China, which was approved by the Medical Ethics Committee of Guangzhou Medical University (ID: 202305001). Informed consent was obtained from both participants and their guardians prior to participation. To provide a clear visual representation of our participant selection process, we have included a participant flow chart as **Figure 1**. In accordance with the verbal reports and case certification provided by teachers and parents, the inclusion criteria were: (1) participants agreed to participate in the study, and informed verbal and written consent was obtained from parents; (2) participants were aged between 11 and 15 years; (3) participants had normal reading ability; (4) participants had no mental or psychological disorders and had normal physical and neurological examinations; (5) participants had lived with their parents since childhood. Questionnaires that met the exclusion criteria, as determined by the research team during the data cleaning phase, were excluded from the study. The exclusion criteria included the following: (1) completion time less than 100 seconds; and (2) presence of outliers, such as the number of duplicate items was greater than 5 or failure of the randomized test question (e.g., “I forgot how to spell my name”).

A total of 1,075 junior high school students completed the questionnaire, and 1,067 valid questionnaires were ultimately included, resulting in an effective response rate of 99.26%. The sampling participation rate is 44.46%, with 2,400 students enrolled. The 1,067 participants (496 boys, representing 46.49%, and 571 girls, representing 53.51%) had an average age of 13.12 years. In the entire school, the ratio of boys to girls is 84% (1,096 boys and 1,304 girls). In the sample, the ratio of boys to girls is 87% (496 boys and 571 girls). The grades were concentrated in seventh grade (46.58%) and eighth grade (48.92%). Most participants came from non-single-child families (81.91%), and a significant portion of their fathers (41.52%) or mothers (42.74%) had a junior high school education. A substantial number of participants (42.55%) reported satisfaction with their peer relationships. Details are shown in **Table 1**.

**Table 1:** Basic information of participants (*N* = 1067).

Variables		*N* (%) or Mean ± SD
Age		13.12 ± 0.79
Gender	Male	496 (46.49)
	Female	571 (53.51)
Grade	Seventh	497 (46.58)
	Eighth	522 (48.92)
	Ninth	48 (4.50)
Only-child	No	874 (81.91)
	Yes	193 (18.09)
Education level of father	Primary school degree or below	46 (4.31)
	Junior high school degree	443 (41.52)
	High school or technical secondary school degree	356 (33.36)
	Junior college degree	143 (13.40)
	Bachelor degree or above	79 (7.40)
Education level of mother	Primary school degree or below	75 (7.03)
	Junior high school degree	456 (42.74)
	High school or technical secondary school degree	305 (28.58)
	Junior college degree	159 (14.90)
	Bachelor degree or above	72 (6.75)
Student relationship satisfaction	Very satisfied	68 (6.37)
	Relatively satisfied	83 (7.78)
	Normal	229 (21.46)
	Less satisfied	454 (42.55)
	Very dissatisfied	233 (21.84)

### 2.2 Measures

#### 2.2.1 Simplified Parenting Styles Scale (SPSS)

The Simplified Parenting Styles Scale (SPSS) revised by Jiang (39) includes 42 items, with 21 items each for fathers and mothers. Adolescents are invited to complete the scale in order to indicate the style they perceive in their parents. The scale was originally developed by Swiss scholars to examine the parenting styles of parents by having the subjects recall the way their parents treated them during their growth, including four core dimensions: rejection, emotional warmth, overprotection, and favoritism (40). The Chinese version of the scale was initially revised in 1993 and has since been employed with considerable frequency (41). However, the scale had issues like excessive items, inconsistent item numbers for parents, and the dimension of favoring subject is less relevant due to China’s one-child policy (39). So, it has been introduced and revised to address these shortcomings, yielding a reliable and widely used instrument. It comprises three dimensions: rejection, warmth, and overprotection. The warmth dimension can be classified as a positive parental style, while the rejection and overprotection dimensions can be classified as a negative parental style. The scale uses a four-point Likert scale, where 1 indicates “never” and 4 indicates “always.” Specific items in the scale include: “My father often treats me in a way that embarrasses me,” “My mother often treats me in a way that embarrasses me,” “My father punishes me for even small mistakes,” and “My mother punishes me for even small mistakes.” The Cronbach’s α coefficient for the total scale is 0.86, and the Cronbach’s α coefficients for each dimension are: rejection 0.88 (father) and 0.89 (mother); warmth 0.94 (father) and 0.94 (mother); overprotection 0.69 (father) and 0.68 (mother).

#### 2.2.2 Bystander Behavior Scale (BBS)

The Bystander Behavior Scale (BBS) developed by Ma J (42) includes 19 items, divided into three dimensions: bully promoter, victim protector, and outsider. The earliest classification of bystanders’ roles in school bullying behavior was based on the Participant Role Questionnaire (PRQ) compiled by Salmivalli (43). It divided bystanders’ roles into four types: reinforcers, protectors, assistants, and outsiders. Zhang revised the Chinese version of the PRQ, dividing bystander roles into collaborative bullies, instigators, outsiders, and protectors (44). Later, Wu divided bystanders’ behavioral roles into three categories: reinforcers of bullying, defenders of bullying, and outsiders (45). Ma J applied this scale in the investigation of school bullying in the Chinese context. By deleting items with relatively low correlation indexes, the BBS was finally formed (42). The BBS uses a five-point Likert scale, where 1 indicates “completely disagree” and 5 indicates “completely agree.” Specific items in the scale include: “I would help the bullies bully others,” “I would call others to join in the bullying,” and “I would help the bully hide the fact that they bullied others.” The Cronbach’s α coefficients for the total scale and the three dimensions are 0.73, 0.89, 0.88, and 0.76, respectively.

#### 2.2.3 Basic Empathy Scale (BES)

The Basic Empathy Scale (BES) developed by Jollife (46), includes 20 items, comprising two dimensions: cognitive empathy (9 items) and affective empathy (11 items). This scale was compiled based on Cohen’s definition of empathy as “the understanding and sharing in another’s emotional state or context” (47). It overcame issues such as the confusion between empathy and transference in the Hogan Empathy Scale (HES), Questionnaire Measure of Emotional Empathy (QMEE), and Interpersonal Reactivity Index (IRI), as well as insufficient applicability to different populations or the lack of cognitive/affective empathy dimensions (46). Chinese scholars collaborated with Jollife to translate it into Chinese in 2011. The BES was first applied in Chinese adolescent groups and has reliable reliability, validity and applicability (48). The scale uses a five-point Likert scale, where 1 indicates “strongly disagree” and 5 indicates “strongly agree.” Higher scores indicate stronger empathy. Specific items in the scale include: “My friend’s emotions do not affect me much,” “I often feel sad after being with a friend who is sad,” and “When a friend does well, I feel their joy.” The Cronbach’s α coefficient for this scale is 0.80.

#### 2.2.4 Family Violence Scale (FVS)

Family Violence Scale (FVS) is adapted from the scale intimate partner violence (IPV), which was measured using a self-compiled five-point scale ranging from 0 (never) to 4 (always). The IPV scale encompasses two dimensions: physical and mental violence. Physical violence includes both physical IPV and sexual IPV, assessed through two behaviorally specific items (49), such as “Family members or boy/girlfriends punched me with a fist or an object” and “Family members or boy/girlfriends had body contact or sex with me when I did not want to.” Mental violence is based on the emotionally abusive behaviors proposed by Maiuro (50) and includes three dimensions: (1) denigrating damage to self-esteem, e.g., “Family members or friends compared me with others or accused me in public, making me embarrassed and unconfident”; (2) passive-aggressive withholding of emotional support and nurturance, e.g., “When I was not feeling well or in a bad mood, my Family members or friends didn’t care about me”; and (3) restricting personal territory and freedom, e.g., “Family members or friends checked my phone or decided my dressing and relationships.” The total score of the IPV scale ranges from 0 to 20, with higher scores indicating more severe IPV experienced by the subject. The scale of the Cronbach ‘s alpha coefficient is 0.84.

### 2.3 Data analysis

This study used SPSS 26 (51) and MPlus 8.1 (52) to analyze the data. The specific steps were as follows: (1) Harman’s single-factor test was used to check for common method bias; (2) Descriptive statistical analysis was conducted to analyze the basic characteristics of the participants; (3) Pearson correlation analysis was used to examine the correlations between the scales; (4) The mediation effect between parental styles, basic empathy, and bystander behaviors, as well as the moderating role of family violence, was tested using the mediation and moderation analysis code (PROCESS Model 59) in MPlus.

## 3 Results

### 3.1 Common method bias

In the common method bias test, all items from the parental styles, basic empathy, family violence, and bystander behavior scales were included. The results showed that 17 factors had eigenvalues greater than 1. The first factor explained 16.65% of the variance, which is less than the critical value of 40%. Therefore, there was no severe common method bias in this study.

### 3.2 Correlation analysis

**Table 2** shows the means and standard deviations of parental styles, basic empathy, bystander behavior, and family violence, as well as the correlations between the scales. The results revealed a modest positive correlation between an affirming parenting style and the manifestation of basic empathy and victim protection behaviors, with statistically significant coefficients (*r* = 0.29, *p* < 0.01 for both). Furthermore, an affirming parenting style exhibited a moderate negative correlation with instances of family violence, which was also statistically significant (*r* = -0.38, *p* < 0.01). Conversely, a negative parenting style correlated positively, albeit weakly, with bully promotion behaviors, and strongly with family violence, with both correlations being statistically significant (*r* = 0.12, *p* < 0.01 for bully promotion behaviors; *r* = 0.62, *p* < 0.01 for family violence). Although the association between basic empathy and victim protection behaviors showed a weak positive correlation (*r* = 0.33, *p* < 0.01), and the correlation with bully promotion behaviors was weakly negative (*r* = -0.10, *p* < 0.01), both reached statistical significance.

**Table 2:** Descriptive statistics and correlation of each scale (*N* = 1067).

Variables	M ± SD	1	2	3	4	5	6	7
1.Positive parental style	18.61 ± 5.79	1						
2.Negative parental style	12.36 ± 2.81	-0.19**	1					
3.Basic empathy	69.20 ± 10.30	0.29**	0.04	1				
4.Bully promoter	10.36 ± 3.72	-0.06	0.12**	-0.10**	1			
5.Victim protector	18.54 ± 5.58	0.29**	0.06	0.33**	-0.10**	1		
6.Outsider	12.27 ± 4.56	0.03	0.11**	0.03**	0.16**	-0.01	1	
7.Family Violence	7.59 ± 3.69	-0.38**	0.62**	-0.04	0.13**	0.01	0.10**	1

^*^ *p* < 0.05, ^**^ *p* < 0.01, ^***^ *p* < 0.001.

### 3.3 Mediation effect analysis

Multifactor logistic regression was used to further examine the causal relationships between parental styles and bystander behaviors. The results are shown in **Table 3**. After controlling for age, gender, the education level of the parents and only child as confounding variables, a positive parental style still had a significant positive predictive effect on basic empathy and victim protector behaviors (*β* = 0.293, *p* < 0.001; *β* = 0.208, *p* < 0.001). A negative parental style significantly positively predicted bully promoter behaviors (*β* = 0.126, *p* < 0.001). Basic empathy negatively predicted bully promoter behaviors (*β* = -0.098, *p* < 0.01) and positively predicted victim protector behaviors (*β* = 0.249, *p* < 0.001).

**Table 3:** Multiple logistic regression analysis (*N* = 1067).

Variables	Basic empathy						Bully promoter			Victim protector		
	Model 1			Model 2			Model 3			Model 4		
	*β*	*SE*	*t*	*β*	*SE*	*t*	*β*	*SE*	*t*	*β*	*SE*	*t*
Age	0.007	0.375	0.244	-0.025	0.391	-0.839	0.008	0.144	0.278	-0.110	0.199	-3.910^***^
Gender	0.242	0.588	8.486^***^	0.235	0.615	7.877***	-0.021	0.232	-0.662	0.096	0.323	3.338^**^
Basic empathy							-0.098	0.011	-3.153**	0.249	0.016	8.282^***^
Positive parental style	0.293	0.055	9.785^***^							0.208	0.028	7.088^***^
Negative parental style				0.048	0.109	1.593	0.126	0.040	4.162^***^			
*R^2^*	0.140			0.057			0.041			0.173		
*F*	57.652^***^			21.527^***^			7.224^***^			55.578^***^		

^*^ *p* < 0.05, ^**^ *p* < 0.01, ^***^ *p* < 0.001.

The mediation effect of basic empathy between positive parental style and victim protector behaviors, as well as negative parental style and bully promoter behaviors, was tested. After 5000 bootstrap samples, the following results were found:

First, the confidence interval for the simple mediation effect of positive parental style → basic empathy → victim protector behaviors did not include 0, indicating that basic empathy partially mediates this path.

Second, the confidence interval for the simple mediation effect of negative parental style → basic empathy → bully promoter behaviors included 0, indicating that the mediation effect of basic empathy in this path is not significant. The mediation effect paths are shown in **Table 4**.

**Table 4:** The mediation effect of each path (*N* = 1067).

Path	Effect	BootSE	BootLLCI	BootULCI
Positive parental style→Victim protector	0.201	0.028	0.145	0.257
Positive parental style→Basic empathy→Victim protector	0.070	0.011	0.050	0.093
Total effect	0.271	0.028	0.217	0.326
Negative parental style→Bully promoter	0.167	0.040	0.088	0.246
Negative parental style→Basic empathy→Bully promoter	-0.006	0.005	-0.017	0.002
Total effect	0.161	0.040	0.082	0.240

### 3.4 Moderation effect analysis

To test whether family violence moderates the mediation effect of positive parental style, basic empathy, and victim protector behaviors, family violence was included as a moderating variable. As shown in **Table 5**, the interaction term of positive parental style and family violence significantly negatively predicted basic empathy and victim protector behaviors (*β* = -0.075, *p* < 0.001; *β* = -0.025, *p* < 0.01). However, the interaction term of basic empathy and family violence did not significantly predict victim protector behaviors (*β* = 0.004, *p* > 0.05). In other words, family violence significantly weakens the impact of positive parental style on basic empathy and victim protector behaviors.

**Table 5:** The moderating effect of family violence on positive parenting style and empathy (*N* = 1067).

Variables	Basic empathy			Victim protector		
	Model 1			Model 2		
	*β*	*SE*	*t*	*β*	*SE*	*t*
Age	0.084	0.370	0.227	-0.805	0.197	-4.084***
Gender	4.502	0.584	7.706***	0.932	0.320	2.914**
Positive parental style	0.533	0.055	9.785***	0.249	0.031	8.076***
Family violence	0.052	0.088	0.593	0.146	0.047	3.095**
Positive parental style×Family violence	-0.075	0.014	-5.525***	-0.025	0.007	-3.415**
Basic empathy				0.121	0.016	7.400***
Basic empathy×Family violence				0.004	0.004	1.055
*R^2^*	0.168			0.196		
*F*	42.795***			36.847***		

^*^ *p* < 0.05, ^**^ *p* < 0.01, ^***^ *p* < 0.001.

**Figure 2:**
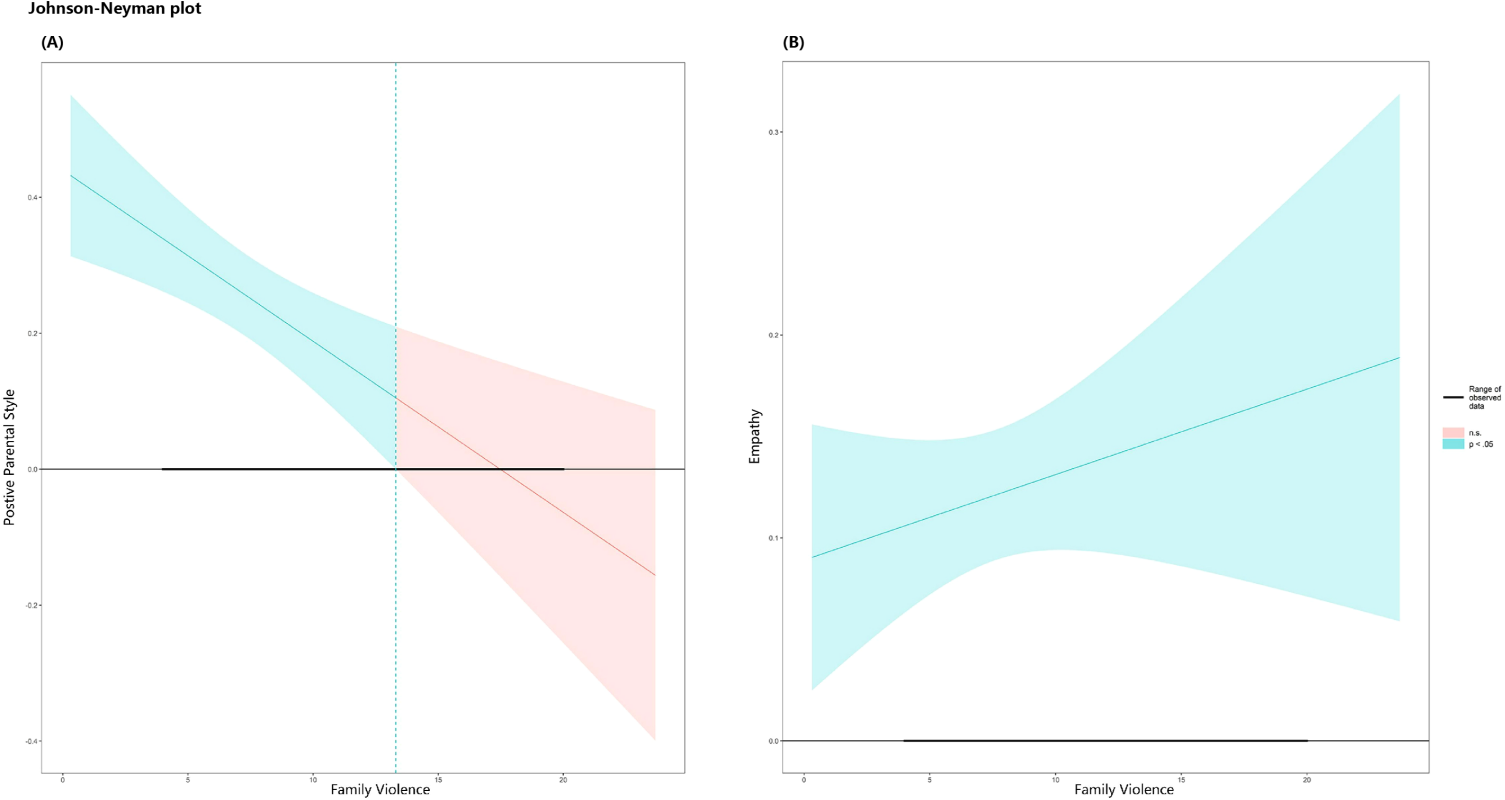
Johnson-Neyman plot for the interaction effect of family violence on both positive parental style and empathy in relation to victim protector behaviors. **(A)** The interaction effect of family violence on positive parental style and victim protector behaviors. **(B)** The interaction effect of family violence on empathy and victim protector behaviors. The green line and area represent the values and confidence intervals of significant moderating variables; Red lines and area represent the values and confidence intervals of non-significant moderating variables.

**Figure 3:**
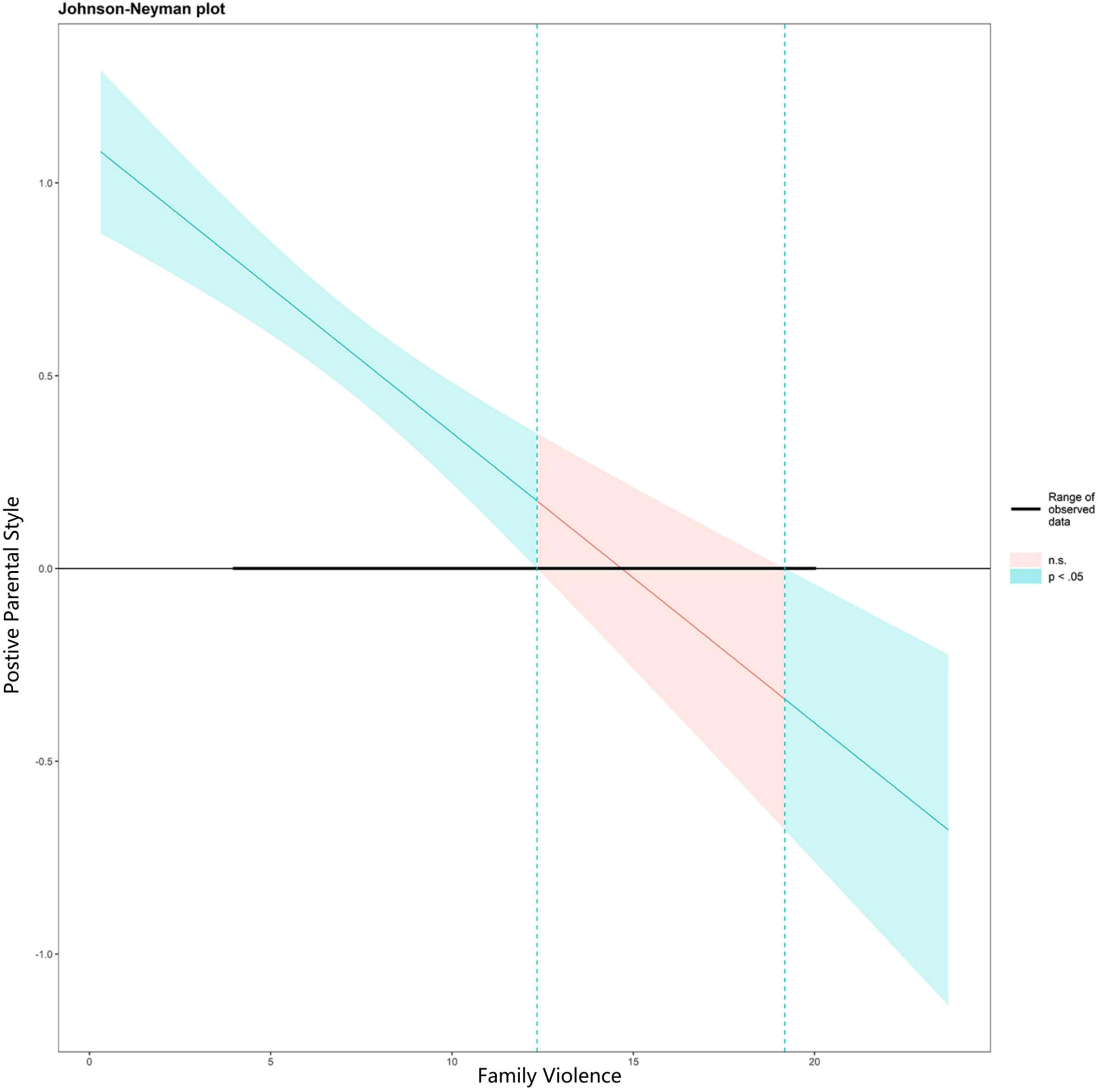
Johnson-Neyman plot for the interaction effect of family violence on positive parental style and empathy. The green line and area represent the values and confidence intervals of significant moderating variables; Red lines and area represent the values and confidence intervals of non-significant moderating variables.

To fully reflect the moderated mediation effect of family violence, the Johnson-Neyman method was used to calculate the 95% confidence intervals and specific values of the significant regions. The conditional indirect effects at different continuous values of the moderating variable, family violence, were graphically represented. As shown in **Figures 2**, **3**, the effect of positive parental style on victim protector behaviors is significant when the value of family violence is less than 13.31; the effect of positive parental style on basic empathy is significant when the value of family violence is outside the range of [12.35, 19.18]; and the effect of basic empathy on victim protector behaviors is significant across all values of family violence.

**Figure 4:**
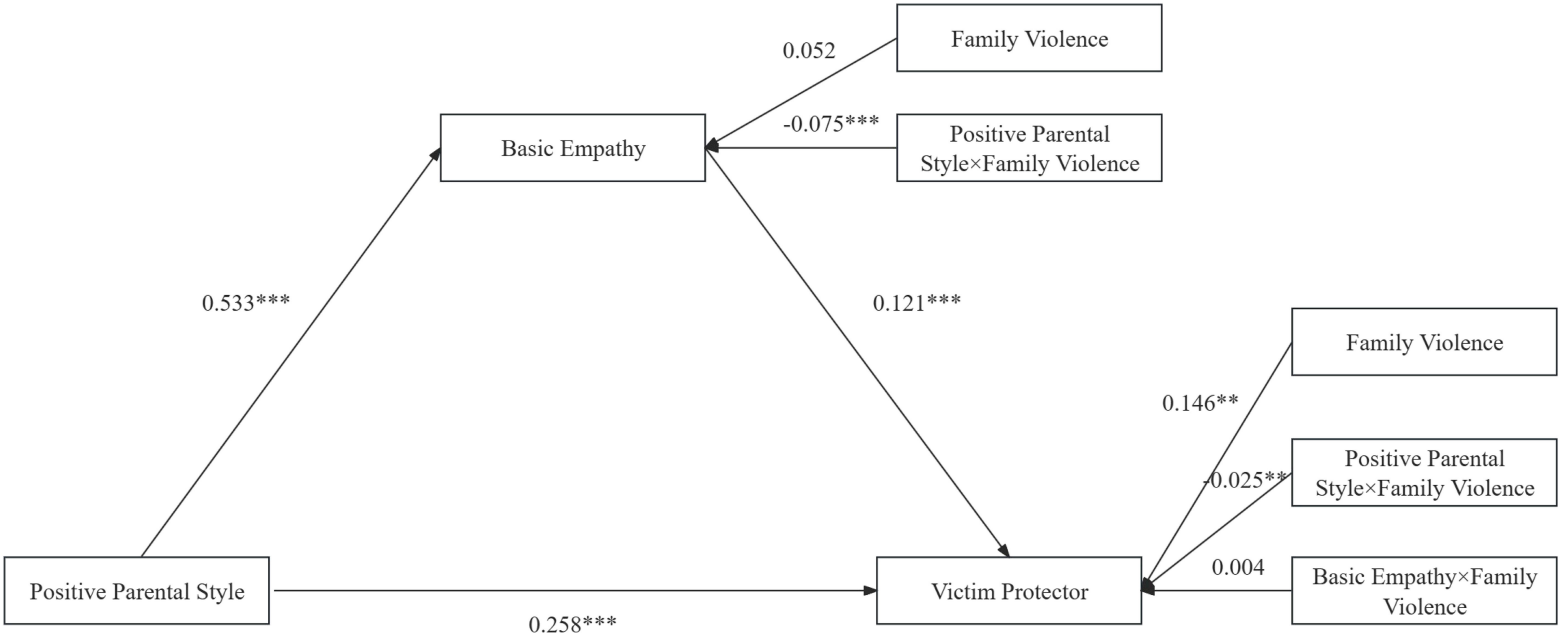
Moderating meditation effect pattern. **p < 0.01, ***p < 0.001.

According to **Table 6**, the confidence intervals for all paths do not include 0, indicating that the indirect effects are significant regardless of the level of family violence. The moderated mediation model is shown in **Figure 4**.

**Table 6:** The moderated mediating effect (N = 1067).

Variables		Effect	BootSE	BootLLCI	BootULCI
Path1	M-1SD	0.342	0.040	0.263	0.420
	M+1SD	0.156	0.042	0.073	0.239
Path2	M-1SD	0.811	0.070	0.674	0.948
	M+1SD	0.256	0.078	0.102	0.409
Path3	M-1SD	0.106	0.022	0.062	0.149
	M+1SD	0.137	0.022	0.094	0.180
Path4	M-1SD	0.086	0.021	0.043	0.126
	M+1SD	0.065	0.011	0.012	0.063

## 4 Discussion

This study aims to explore the effects of both positive and negative parenting styles on adolescents’ basic empathy and protective behaviors toward victims, while also examining the moderating role of domestic violence. The results support Hypotheses 1 and 2, indicating that positive parenting is significantly positively correlated with basic empathy and protective behaviors, whereas negative parenting is significantly positively correlated with bullying-supportive behaviors and domestic violence (53, 54). The mediation analysis further validated the establishment of Hypothesis 3. The results indicated that, after controlling for confounding variables such as age, gender, parents’ education level, and only-child status, positive parenting was associated with higher levels of basic empathy and protective behaviors, while negative parenting was associated with higher levels of bullying-supportive behaviors. Additionally, the study found a negative correlation between basic empathy and bullying-supportive behaviors, and a positive correlation between basic empathy and protective behaviors (53, 55). The moderation analysis partially supports Hypothesis 4, showing that domestic violence significantly weakens the positive impact of positive parenting on both basic empathy and protective behaviors (56, 57). However, the interaction between basic empathy and domestic violence did not reach statistical significance in predicting protective behaviors.

### 4.1 Enhancing the impact of family education on basic empathy

Our research indicates that there is a significant positive relationship between a positive parental style and adolescents’ basic empathy. This finding highlights the crucial role of the family environment in shaping adolescents’ socio-emotional development. A positive parental style not only includes emotional support, active communication, and modeling behavior but also involves sensitivity and responsiveness to children’s emotional needs (58, 59).

On one hand, parents, as their children’s first teachers, set important examples through their behaviors and attitudes (60). During daily interactions, adolescents internalize the empathy skills demonstrated by their parents through observation and imitation (61). For instance, when parents show compassion and helping behaviors toward others in distress, adolescents are likely to emulate these behaviors, thereby enhancing their own empathy skills.

On the other hand, a positive parental style creates a safe and supportive emotional environment (59). In such an environment, adolescents feel understood and accepted, which is a vital foundation for developing empathy (62). The findings of Luo et al. indicate that when adolescents perceive unconditional support and understanding from their parents, they are more likely to exhibit sensitivity and responsiveness to others’ emotions and needs (63).

Furthermore, emotional education plays a significant role in a positive parental style (64). Through daily interactions, parents teach their children how to recognize and manage their own emotions, helping them understand and respond to the emotional needs of others (65). This emotional education not only directly promotes the development of adolescents’ empathy but also enhances their self-efficacy in social interactions, making them more confident in forming positive relationships with others.

Therefore, to foster adolescents’ empathy, parents should adopt a positive parental style by providing emotional support, engaging in active communication, modeling empathic behaviors, and being sensitive and responsive to their children’s emotional needs. Creating a safe and supportive emotional environment and incorporating emotional education into daily interactions will help adolescents identify, manage, and respond to emotional needs, thereby enhancing their socio-emotional development and self-efficacy.

### 4.2 The crucial role of basic empathy in bystander behavior

This study has found that adolescents with high basic empathy are more inclined to engage in victim protector behaviors. This result from our study underscores the central role of empathy in motivating prosocial behaviors. Individuals with high basic empathy are more likely to experience emotional arousal when witnessing others in distress, and this emotional arousal can be translated into the motivation to help others (66). Empathy not only helps individuals understand others’ emotions but also plays a critical role in their behavioral decision-making process. On one hand, individuals with high basic empathy are more attuned to others’ needs and are therefore more likely to take action to alleviate others’ distress (67). For instance, in school bullying incidents, bystanders with high basic empathy are more likely to intervene to stop the bullying or support the victim. Additionally, individuals with high basic empathy typically possess stronger social skills, which enable them to establish and maintain healthy interpersonal relationships within groups (68). These social networks provide them with opportunities and support to engage in bystander intervention behaviors. Through these social networks, they can access more resources and encouragement, thereby increasing their confidence in taking prosocial actions.

Therefore, educational and psychological interventions should focus specifically on fostering adolescents’ basic empathy. This approach not only helps reduce bullying behaviors but also promotes a more prosocial atmosphere within the school environment. Research indicates that enhancing empathy can effectively lower the incidence of bullying and increase bystanders’ support for victims (69, 70). Additionally, implementing interventions centered around empathy can further improve the social climate in schools, enhancing understanding and cooperation among students and thereby reducing the occurrence of bullying behaviors (71).

### 4.3 The bridging role of emotional cultivation

This study indicates that basic empathy plays a crucial bridging role between a positive parental style and victim protector behaviors, emphasizing the importance of emotional cultivation in family education. Specifically, a positive parental style can indirectly promote victim protector behaviors in adolescents by enhancing their basic empathy. This finding from our study suggests that parents should not only focus on behavioral norms in daily upbringing but also pay attention to the emotional development of adolescents. By guiding adolescents to understand and experience others’ emotions, parents can effectively cultivate prosocial behaviors in their children (68). This emotional guidance helps adolescents better identify and respond to others’ needs in social situations and can inspire protective and helping behaviors, thereby creating a more positive impact on society (72).

### 4.4 The negative impact of family violence on emotional and behavioral development

This study reveals that family violence significantly weakens the positive impact of a positive parental style on basic empathy and protective behaviors. This result reveals the profound effects of a violent environment on children’s psychological and behavioral development. A study found that adolescents in violent family environments typically exhibit lower basic empathy (73). This is because prolonged exposure to violence and conflict can lead adolescents to develop self-protection mechanisms, reducing their focus on and understanding of others’ emotions. Despite the presence of a positive parental style, family violence still negatively affects adolescents’ emotional understanding and social behaviors.

Family violence directly damages adolescents’ emotional security and behavioral norms (74–76). A positive parental style usually emphasizes warmth, support, and effective communication, but violent behaviors convey the opposite message, leading adolescents to feel fear and distrust toward their family environment. This emotional insecurity severely undermines the effectiveness of positive parenting, hindering adolescents from gaining positive emotional and behavioral influences.

According to social learning theory (77, 78), adolescents learn how to handle conflicts and express emotions by observing and imitating their parents’ behaviors. If they witness or experience family violence, they are more likely to learn to solve problems through violence and aggression rather than understanding and empathy. Adolescents in violent environments may develop unhealthy emotional processing and coping mechanisms (79). Prolonged exposure to violence can lead to stress responses such as emotional numbness, anxiety, and depression (80, 81), which can weaken their emotional understanding and empathy in social situations, affecting their interactions with others.

This finding highlights the necessity of addressing family violence when intervening in family education and mental health issues. Social services and mental health professionals need to work together to provide multi-level support and interventions to reduce the impact of family violence on adolescent development.

### 4.5 Constructing multi-level intervention strategies

The research findings provide important insights for designing multi-level anti-bullying intervention programs, emphasizing that intervention strategies need to go beyond the school environment and penetrate various aspects of the family environment. Firstly, schools should continue to implement direct anti-bullying measures, but more importantly, intervention strategies should also include improving parental styles, particularly addressing issues of family violence. Through family education training, parents can enhance their parenting skills and emotional management abilities, thereby creating a healthier family environment. Additionally, the cultivation of basic empathy should not only be carried out among students but also promoted among family members to enhance understanding and support within the family. Interventions targeting family violence are also indispensable, as these measures can directly reduce conflicts and violent behaviors within the family, providing a safer growth environment for children and reducing their likelihood of bullying or being bullied at school.

In summary, multi-level intervention strategies should cover family education, basic empathy cultivation, and family violence intervention, forming a comprehensive and systematic intervention framework. To support the effectiveness of these strategies, evidence shows that incorporating parental components into school-based anti-bullying programs can significantly reduce bullying behaviors (82). Furthermore, specific programs like the KiVa Anti-bullying Program have demonstrated significant positive effects in various school grades, reinforcing the importance of implementing effective school-based interventions (83). These strategies can not only reduce bullying behaviors but also promote students’ social adaptation and psychological health development. To achieve these goals, schools, families, and communities need to work closely together to form a supportive ecosystem that creates a safe and healthy growth environment for students.

## 5 Limitations and future directions

This study delves into the combined effects of parental styles, basic empathy, and family violence on bystander behaviors, providing significant insights in the related field. However, there are certain limitations. Firstly, due to the cross-sectional design, causal relationships between variables cannot be inferred. Future studies could further verify this mediation model through longitudinal designs. Secondly, participants were only from three middle schools in southern China, which limits the geographic representativeness of the sample, making it difficult to generalize the findings to bystander behaviors in schools across other regions of China. Additionally, the survey tools used in this study have specificity, and using different assessment scales might lead to different mediation structures. It is also important to note that the instrument for measuring bystander behavior is a self-report questionnaire. There is evidence suggesting that self-report measures, especially on sensitive topics like bullying, are subject to high social desirability bias (84, 85), which should be considered. Therefore, future research should focus on further improving these measurement tools to minimize the impact of social desirability bias and enhance data accuracy. Additionally, the tools currently used to measure parenting styles may not fully capture the complexity of parental behaviors and attitudes, potentially leading to biased or incomplete data (86, 87). Researchers should work to optimize these measurement methods to ensure the accuracy of study results. Future research should expand the geographical representation of the sample and increase the sample size to better validate these findings. These studies will help in a deeper understanding of the related mechanisms. Moreover, future research should explore various other factors that might influence adolescent behaviors, which could have profound effects on adolescents either directly or indirectly.

Our research has several practical implications. Firstly, schools can help students benefit from bystander behaviors and reduce bullying behaviors by promoting a positive school atmosphere and cultivating effective interpersonal skills. Secondly, at the adolescent level, the cultivation of basic empathy is crucial as it can prompt them to engage in victim protector behaviors and promote prosocial behaviors. Finally, in the family environment, parents need to cultivate healthy parent-child attachment styles and address issues of family violence. By improving parenting skills through family education training, they can create a healthy family environment. In summary, multi-level intervention strategies should encompass family education, basic empathy cultivation, and family violence intervention to form a comprehensive intervention framework that promotes students’ social adaptation and psychological health development.

## 6 Conclusion

(1) A positive parental style significantly enhances adolescents’ basic empathy, improving their emotional regulation and social skills through warmth and support. (2) Adolescents with high basic empathy are more inclined to engage in victim protector behaviors, highlighting the central role of empathy in motivating prosocial behaviors. (3) Positive parental styles indirectly promote victim protector behaviors in adolescents by enhancing their basic empathy, emphasizing the importance of emotional cultivation. (4) Family violence weakens the positive impact of positive parental styles on basic empathy and protective behaviors, damaging adolescents’ emotional security and behavioral norms. (5) Designing multi-level intervention strategies should encompass family education, basic empathy cultivation, and family violence intervention to promote students’ social adaptation and psychological health development.

## Data Availability

The original contributions presented in the study are included in the article/**Supplementary Material**. Further inquiries can be directed to the corresponding author.
