# Supplementary material for: Exploring the interplay of parenting styles, basic empathy, domestic violence, and bystander behavior in adolescent school bullying: a moderated mediation analysis
Source: Front Psychiatry. 2024 Sep 9;15:1452396. doi: 10.3389/fpsyt.2024.1452396 (PMC11416980; doi:10.3389/fpsyt.2024.1452396)
Supplement: Supplementary file 1 [file Table1.docx]

A Survey of Exploring the Interplay of Parenting Styles, Basic Empathy, Domestic Violence and Bystander Behavior in Adolescent School Bullying

Dear students,

hello!

We are students from the Department of Applied Psychology at Guangzhou Medical University. We are conducting a questionnaire survey on adolescent behavior. Thank you very much for taking the time to fill out this questionnaire. Your answers will be used for scientific research only. Your data will be kept strictly confidential, so please feel free to answer.

There is no right or wrong answer for all the following questions. Please answer them carefully and independently according to the actual situation. Thank you very much for your cooperation!

1. Your gender: [single choice] *

- Male
- Female

1. Your age [fill in the blanks] *
2. Your grade: [single choice] *

- Seventh
- Eighth
- Nineth

1. Are you an only-child? [single choice] *

- Yes
- No

1. Father's education level: [single choice] *

- Primary school and below
- Junior high school
- High school/technical secondary school
- Junior college
- Bachelor degree or above

1. Mother's education level: [single choice] *

- Primary school and below
- Junior high school
- High school/technical secondary school
- Junior college
- Bachelor degree or above

1. Satisfaction with current relationship with classmates [single choice] *

- Very satisfied
- Relatively satisfied
- Normal
- Less satisfied
- Very dissatisfied

8. Please read the following description carefully, judge how it fits with your idea or practice, and choose the option that best fits your idea or practice. [Matrix multiple choice] *

|  | Totally out of line | More inconsistent | Uncertain | More in line | A perfect match |
| --- | --- | --- | --- | --- | --- |
| I will help the bully escape if the teacher finds out. |  |  |  |  |  |
| I help bullies spread the word about bullying. |  |  |  |  |  |
| I will call others along to help the bully spread the word about bullying. |  |  |  |  |  |
| I will tell victims how to get help if they are being bullied. |  |  |  |  |  |
| I would ask other viewers to help the victim. |  |  |  |  |  |
| I alert the victim when I know bullying is likely to occur. |  |  |  |  |  |
| I will help victims report to teachers. |  |  |  |  |  |
| I will help victims stand up to bullies. |  |  |  |  |  |
| I will not help anyone. |  |  |  |  |  |
| I just stood by and watched, trying not to relate to the bullying. |  |  |  |  |  |
| I will stay away from bullying venues. |  |  |  |  |  |
| I will persuade others to stay away from bullying venues. |  |  |  |  |  |
| I took one look and left quickly. |  |  |  |  |  |
| I will stay away from the bullying arena. |  |  |  |  |  |
| I would advise others to stay out of the bullying arena. |  |  |  |  |  |
| I took a look and hurried away. |  |  |  |  |  |

9. Please answer the following questions based on your experience. Your father and mother may have raised you in the same way or in different ways. Please answer truthfully. Choose only one answer for each question. [Matrix multiple choice] *

|  | Never | Occasionally | Often | Always | Never |
| --- | --- | --- | --- | --- | --- |
| My father often treats me in a way that embarrass es me. |  |  |  |  |  |
| My mother often treats me in a way that embarrass es me. |  |  |  |  |  |
| My father punishes me for even small mistakes. |  |  |  |  |  |
| My mother punishes me for even small mistakes. |  |  |  |  |  |
| My father often criticizes me in front of others for being lazy and useless. |  |  |  |  |  |
| My mother often criticizes me in front of others for being lazy and useless. |  |  |  |  |  |
| My father often punishes me more than I deserve. |  |  |  |  |  |
| My mother often punishes me more than I deserve. |  |  |  |  |  |
| My father often loses his temper with me without me knowing why. |  |  |  |  |  |
| My mother often loses her temper with me without me knowing why. |  |  |  |  |  |
| I am often used as a "scapegoat" or "black sheep" by my father at home. |  |  |  |  |  |
| I am often used as a "scapegoat" or "black sheep" by my mother at home. |  |  |  |  |  |
| I think my father tries to make my teenage years as meaningful and colorful as possible. |  |  |  |  |  |
| I think my mother tries to make my teenage years as meaningful and colorful as possible. |  |  |  |  |  |
| When things go wrong, I can feel that my father is trying to encourage me and comfort me. |  |  |  |  |  |
| When things go wrong, I can feel that my mother is trying to encourage me and comfort me. |  |  |  |  |  |
| My father always tries to encourage me to excel. |  |  |  |  |  |
| My mother always tries to encourage me to excel. |  |  |  |  |  |
| When I succeed in something I do, I think my father is very proud of me. |  |  |  |  |  |
| When I succeed in something I do, I think my mother is very proud of me. |  |  |  |  |  |
| My father praises me. |  |  |  |  |  |
| My mother praises me. |  |  |  |  |  |
| I feel a warm, thoughtful and affectionate relationship with my father. |  |  |  |  |  |
| I feel a warm, thoughtful and affectionate relationship with my mother. |  |  |  |  |  |
| I can tell by my father's words and expressions that he likes me very much. |  |  |  |  |  |
| I can tell by my mother's words and expressions that she likes me very much. |  |  |  |  |  |
| My father wouldn't allow me to do some of the things other children did because he was afraid something would happen to me. |  |  |  |  |  |
| My mother wouldn't allow me to do some of the things other children did because she was afraid something would happen to me. |  |  |  |  |  |
| When my father asks me to come home, I must explain to him what I did outside. |  |  |  |  |  |
| When my mother asks me to come home, I must explain to her what I did outside. |  |  |  |  |  |
| Does my father always dictate what I should wear or look like? |  |  |  |  |  |
| Does my mother always dictate what I should wear or look like? |  |  |  |  |  |
| I think my father's fear that something might happen to me is exaggerated and overblown. |  |  |  |  |  |
| I think my mother's fear that something might happen to me is exaggerated and overblown. |  |  |  |  |  |
| Does my father put strict limits on what I can and can't do and never give in? |  |  |  |  |  |
| Does my mother put strict limits on what I can and can't do and never give in? |  |  |  |  |  |
| I feel that my father interferes with everything I do. |  |  |  |  |  |
| I feel that my mother interferes with everything I do. |  |  |  |  |  |
| My father often allows me to go where I like without worrying too much. |  |  |  |  |  |
| My mother often allowed me to go where I liked without worrying too much. |  |  |  |  |  |
| I hope my father does not worry too much about what I am doing. |  |  |  |  |  |
| I hope my mother doesn't worry too much about what I am doing. |  |  |  |  |  |

10. Please truthfully answer the following questions based on your life experience. Choose only one answer for each question. [Matrix single choice] *

|  | Totally disagree | Basically disagree | Not sure | Basically agree | Completely agree |
| --- | --- | --- | --- | --- | --- |
| My friend's mood doesn't affect me much. |  |  |  |  |  |
| After spending time with sad friends, you often feel sad yourself. |  |  |  |  |  |
| I can feel my friend's joy when he/she performs well and gets a result. |  |  |  |  |  |
| I get scared when I see some scenes in horror movies. |  |  |  |  |  |
| I get caught up in other people's emotions easily. |  |  |  |  |  |
| It's hard for me to notice when a friend is scared. |  |  |  |  |  |
| I don't get sad when I see someone crying. |  |  |  |  |  |
| Other people's emotions don't bother me at all. |  |  |  |  |  |
| I can usually tell when someone is feeling down. |  |  |  |  |  |
| I can usually tell when a friend is scared. |  |  |  |  |  |
| When I see a sad scene on TV or in a movie, I often feel sad along with it. |  |  |  |  |  |
| I can usually sense people's emotions before they tell me what they're feeling. |  |  |  |  |  |
| It doesn't affect my emotions when I see other people being irritated. |  |  |  |  |  |
| I can usually tell when others are happy. |  |  |  |  |  |
| When my friends with me get scared, I get scared too. |  |  |  |  |  |
| I quickly realize that my friend is angry. |  |  |  |  |  |
| I often get involved in my friends' moods. |  |  |  |  |  |
| My friend's depression doesn't affect me. |  |  |  |  |  |
| I am often unaware of my friends' emotional feelings. |  |  |  |  |  |
| I can hardly tell when a friend is happy. |  |  |  |  |  |

11. Please carefully recall whether similar events have happened in the question stem, and choose the appropriate option according to your actual situation. [Matrix single choice] *

|  | Never | Rarely | Sometimes | Often | Always |
| --- | --- | --- | --- | --- | --- |
| My family members have beaten me directly or hurt me with the help of tools. |  |  |  |  |  |
| When I was in a bad state (not feeling well or in a bad mood), my family didn't care about me. |  |  |  |  |  |
| My family would go through my phone, decide what I was wearing and restrict my interactions. |  |  |  |  |  |
| My family would compare me to others, openly criticize me, and make me feel embarrassed and unsure. |  |  |  |  |  |
